# [^64^Cu]Cu-PEG-FUD peptide for noninvasive and sensitive detection of murine pulmonary fibrosis

**DOI:** 10.1126/sciadv.adj1444

**Published:** 2024-04-10

**Authors:** Hye Jin Lee, Ksenija Bernau, Thomas J. Harr, Zachary T. Rosenkrans, Grace A. Kessler, Kristen Stott, Angie Tebon Oler, Babita Rahar, Terry Zhu, Yadira Medina-Guevara, Nikesh Gupta, Inyoung Cho, Metti K. Gari, Brian M. Burkel, Justin J. Jeffery, Ashley M. Weichmann, Bianca R. Tomasini-Johansson, Suzanne M. Ponik, Jonathan W. Engle, Reinier Hernandez, Glen S. Kwon, Nathan Sandbo

**Affiliations:** ^1^ Pharmaceutical Sciences Division, School of Pharmacy, University of Wisconsin-Madison, 777 Highland Avenue, Madison, WI 53705, USA.; ^2^ Division of Allergy, Pulmonary and Critical Care Medicine, Department of Medicine, School of Medicine and Public Health, University of Wisconsin-Madison, 600 Highland Avenue, Madison, WI 53792, USA.; ^3^ Department of Medical Physics, School of Medicine and Public Health, University of Wisconsin-Madison, 1111 Highland Avenue, Madison, WI 53705, USA.; ^4^ Department of Cell and Regenerative Biology, School of Medicine and Public Health, University of Wisconsin-Madison, 1111 Highland Avenue, Madison, WI 53705, USA.; ^5^ University of Wisconsin Carbone Cancer Center, University of Wisconsin-Madison, 1111 Highland Avenue, Madison, WI, USA.; ^6^ Arrowhead Pharmaceuticals, 502 S. Rosa Rd., Madison, WI 53719, USA.

## Abstract

Idiopathic pulmonary fibrosis (IPF) is a chronic lung disease resulting in irreversible scarring within the lungs. However, the lack of biomarkers that enable real-time assessment of disease activity remains a challenge in providing efficient clinical decision-making and optimal patient care in IPF. Fibronectin (FN) is highly expressed in fibroblastic foci of the IPF lung where active extracellular matrix (ECM) deposition occurs. Functional upstream domain (FUD) tightly binds the N-terminal 70-kilodalton domain of FN that is crucial for FN assembly. In this study, we first demonstrate the capacity of PEGylated FUD (PEG-FUD) to target FN deposition in human IPF tissue ex vivo. We subsequently radiolabeled PEG-FUD with ^64^Cu and monitored its spatiotemporal biodistribution via μPET/CT imaging in mice using the bleomycin-induced model of pulmonary injury and fibrosis. We demonstrated [^64^Cu]Cu-PEG-FUD uptake 3 and 11 days following bleomycin treatment, suggesting that radiolabeled PEG-FUD holds promise as an imaging probe in aiding the assessment of fibrotic lung disease activity.

## Teaser

A radiolabeled PEGylated peptide sensitively detects bleomycin-induced murine pulmonary fibrosis via μPET-based imaging.

## INTRODUCTION

Idiopathic pulmonary fibrosis (IPF) is a chronic and progressive lung disease that results in irreversible scarring within the lungs (*1*, *2*). The natural history of IPF is characterized by a median survival of 2 to 5 years after diagnosis (*3*, *4*). Unfortunately, the few US Food and Drug Administration (FDA)–approved pharmacologic treatments do not reverse fibrosis but only slow disease progression (*5*, *6*). A major clinical problem in the care of patients with IPF is the lack of an established functional biomarker that enables real-time assessment of disease activity. Therefore, development of an imaging biomarker that would enable contemporaneous and noninvasive assessment of disease activity is critical for improved care and prognostication of patients with IPF (*7*).

In the current clinical setting, high-resolution computed tomography (HRCT) is the most widely used imaging technique for diagnosis of IPF (*8*, *9*). While the usual interstitial pneumonia (UIP) radiographic pattern of HRCT is a well-established component of IPF diagnosis, HRCT imaging and spirometry analysis of lung function decline are conventionally based on retrospective analysis and provide limited information on contemporaneous disease activity that is important for appropriate clinical disease management (*10*–*12*). Positron emission tomography (PET) is a noninvasive imaging technique that allows for monitoring of dynamic metabolic or biochemical changes at a molecular level (*13*). Now, there are no FDA-approved PET probes that are routinely used in clinical management of IPF. ^18^Fluorodeoxyglucose (FDG)–PET is used for malignancy diagnosis and staging, as well as other inflammatory conditions that involve glucose transport (*14*, *15*). While some studies demonstrated a possible utility of ^18^FDG uptake in IPF (*16*), the lack of its specificity for IPF pathobiology is a limiting factor, and this approach requires more investigation for its use in disease staging (*17*). Since the pathobiology of IPF is characterized by abnormal fibrotic responses and excessive deposition of extracellular matrix (ECM) molecules (*18*), several probes have been studied that target the cellular and ECM components, such as collagen (*19*), fibroblast activation protein (*20*), and integrins (e.g., α_v_β_6_) (*21*). Nevertheless, there has been limited investigation of a PET probe that can directly target a site of active ECM assembly.

The fibroblastic focus is known as the leading edge of new lung fibrosis, representing the site of de novo ECM deposition (*18*, *22*). Matrix abnormalities in IPF, including the number of fibroblastic foci, correlate with future disease progression (*23*, *24*). Fibronectin (FN) is a glycoprotein that serves as a key scaffolding, wound repair, and signaling molecule (*25*). FN is highly and differentially up-regulated in the fibroblastic foci of the IPF lung where it is deposited into fibrillar matrix, serving as the required scaffold for the formation of other ECM proteins, including collagens (*18*, *22*, *23*, *26*, *27*). Given that FN matrix plays a critical role in the initial phases of ECM deposition, preceding collagen assembly, and has been associated with IPF disease progression (*28*), it has been a target of investigations for inhibiting and detecting fibrotic responses (*29*, *30*).

Functional upstream domain (FUD) is a well-characterized bacterial-derived ~6-kDa peptide with nanomolar binding affinity for the N-terminal 70-kDa domain of FN, a crucial domain for FN matrix assembly (*31*–*37*). FUD peptide, delivered at repeated therapeutic doses, has shown antifibrotic activity in several models of experimental fibroses (*38*–*41*). We have previously demonstrated that PEGylation of FUD (PEG-FUD) peptide prevents its rapid renal elimination and improves its pharmacokinetic properties for in vivo use while preserving its ability to bind FN with nanomolar affinity and disrupt fibrosis developments (*42*–*44*). Recently, our group demonstrated that ^64^Cu-labeled PEG-FUD has a high level of uptake in murine breast cancer (a solid tumor with abundant stromal FN) compared to ^64^Cu-labeled non-PEGylated FUD in μPET/CT imaging (*45*).

Given the tight binding of FUD to the assembly site for FN and the improved pharmacokinetic properties of PEG-FUD in vivo, in this study, we sought to investigate the ability of PEG-FUD to target FN deposition in human IPF tissues ex vivo and during the early time points after bleomycin-induced murine pulmonary injury in vivo (*46*, *47*). For this, we labeled PEG-FUD and the mutated control peptide, PEG-mFUD, which has a compromised binding affinity for FN (*37*), with either Cyanine-5 (Cy5) fluorophore or ^64^Cu radiolabel, and tracked their distribution in human IPF tissue and in the murine model of the disease. We demonstrate that fluorescently labeled PEG-FUD (Cy5-PEG-FUD) specifically targets FN-rich areas of nascent fibrosis in IPF lung tissue compared to a mutated PEGylated peptide control. We additionally find that [^64^Cu]Cu-PEG-FUD–based micro-PET (μPET) imaging results in robust lung uptake 3 and 11 days following bleomycin treatment. These findings set the stage for further development of radiolabeled PEG-FUD PET/CT imaging to serve as a noninvasive and sensitive detection of FN and may be able to contribute to improved assessment of disease activity in human IPF, enabling more efficient therapeutic decision-making and prognostication.

## RESULTS

### Target validation in IPF tissues

**Fig. 1.**
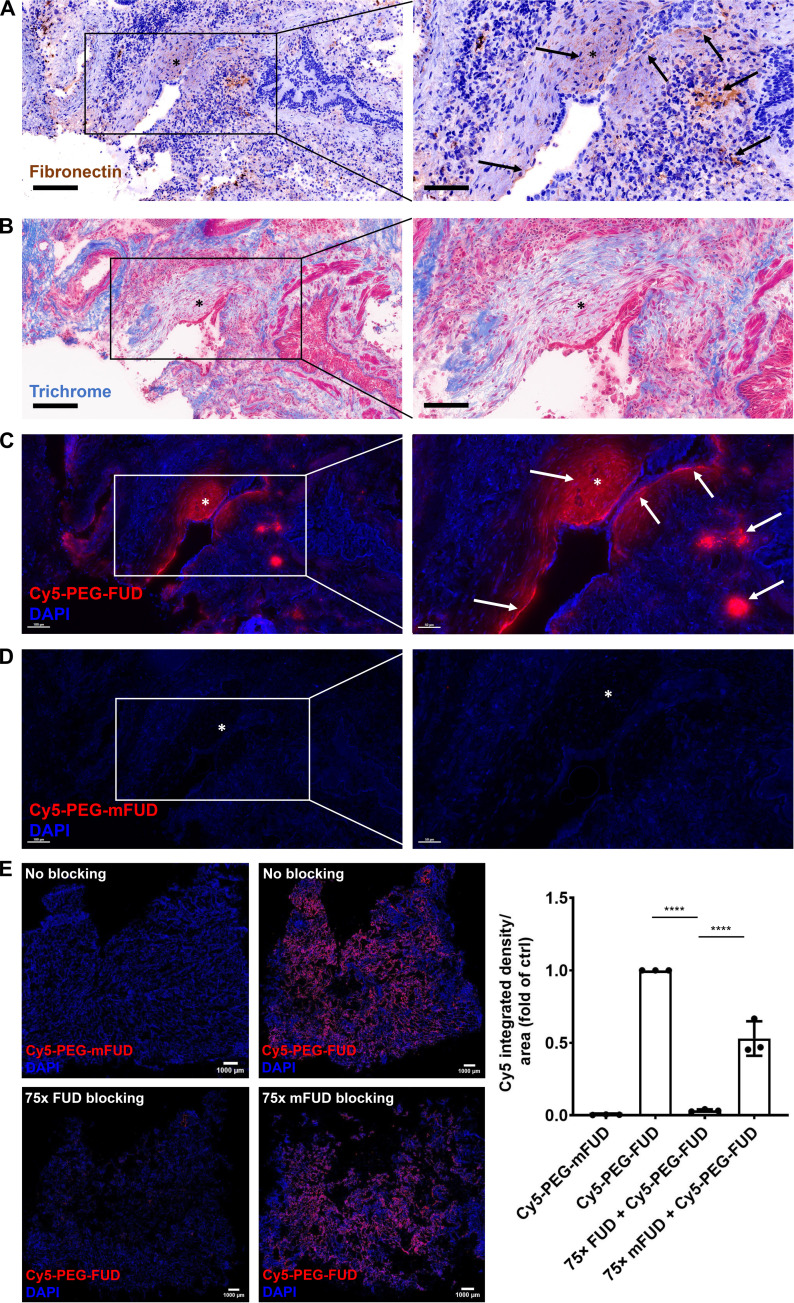
PEG-FUD targets nascent fibrosis in IPF tissues. (**A**) Immunohistochemical staining of human IPF tissue samples against FN and (**B**) with Masson’s trichrome stain (to visualize collagen). (**C**) Cy5-labeled PEG-FUD (Cy5-PEG-FUD) showed localization in IPF tissues in contrast to the (**D**) mutant control (Cy5-PEG-mFUD). Scale bars, 200 μm (left column) and 100 μm (right column) for (A) and (B) and 100 μm (left column) and 50 μm (right column) for (C) and (D). * indicate areas that are morphologically consistent with fibroblastic foci and are digitally zoomed (right column). Arrows point to areas of increased FN staining (A) that correspond to increased Cy5-PEG-FUD binding (C). (**E**) Top row: IPF tissue sections were either stained with Cy5-PEG-mFUD (top left image) or Cy5-PEG-FUD (top right image) without blocking. Bottom row: IPF tissue sections were subjected to blocking with 75× excess of FUD (bottom left image) or mFUD (bottom right image) before being stained with Cy5-PEG-FUD. All tissues were counterstained with DAPI (blue). Scale bars, 1000 μm. Right: Semiquantitative analysis of the blocking studies. Cy5 signal (without DAPI) was normalized to the tissue area and presented as a fold of control. Experiment was repeated *N* = 2 times in the laboratory with *N* ≥ 3 biological replicates. One-way analysis of variance (ANOVA) with Šídák’s multiple comparisons test was used for statistical testing (****P < 0.0001).

FN is a ubiquitous, ECM glycoprotein that is highly and differentially up-regulated in IPF. Previous studies have demonstrated its preferential localization to the fibroblastic focus, the leading edge of active fibrosis (*18*, *22*), suggestive of its critical role in the development of this disease. Similarly, our immunohistochemical staining of IPF lungs revealed high levels of FN and early collagen accumulation in these characteristic structures (Fig. 1, A and B). To determine the spatial localization of PEG-FUD in the fibrotic tissue, we stained these tissues with Cy5 fluorophore-labeled PEG-FUD (Cy5-PEG-FUD) and the identically PEGylated and Cy5-labeled mutant control (Cy5-PEG-mFUD), which differs from FUD by seven amino acid residues and profoundly reduces its binding affinity for FN (*37*, *43*). Our data revealed that Cy5-PEG-FUD preferentially localized to nascent fibrosis by binding to structures morphologically consistent with fibroblastic foci and correlated with the level of FN expression in the consecutive tissue sections (Fig. 1C and fig. S1, E and F). On the other hand, we found no notable targeting of the mutant control to the fibrotic tissue (Fig. 1D and fig. S1D). Tissue incubation with FUD peptide also served to competitively block Cy5-PEG-FUD labeling, evident by more than 95% reduction in Cy5-PEG-FUD binding in this condition, further demonstrating specificity of FUD targeting to FN-rich areas of tissue fibrosis (Fig. 1E). These data suggest that PEG-FUD may serve to target the early profibrotic mechanisms during pulmonary fibrosis disease development.

### Synthesis and ^64^Cu radiolabeling of PEG-FUD

To enable radioactive detection of PEG-FUD by μPET/CT, we initially confirmed that the physicochemical profiles, including purity and binding affinity for FN, for PEG-FUD and PEG-mFUD were similar to those reported previously (*42*, *45*). Radiolabeling of both PEG-FUD and PEG-mFUD conjugates was accomplished within an hour of incubation with ^64^Cu, showing >90% of radiochemical yields for both [^64^Cu]Cu-PEG-FUD and [^64^Cu]Cu-PEG-mFUD. The radio-thin layer chromatography (TLC) image after completion of radiolabeling confirmed that while free ^64^Cu migrated to the top of the TLC plate, radiolabeled [^64^Cu]Cu-PEG-FUD and [^64^Cu]Cu-PEG-mFUD remained at the origin (fig. S2).

### Ex vivo binding of [^64^Cu]Cu-PEG-FUD to bleomycin-induced fibrotic lungs

**Fig. 2.**
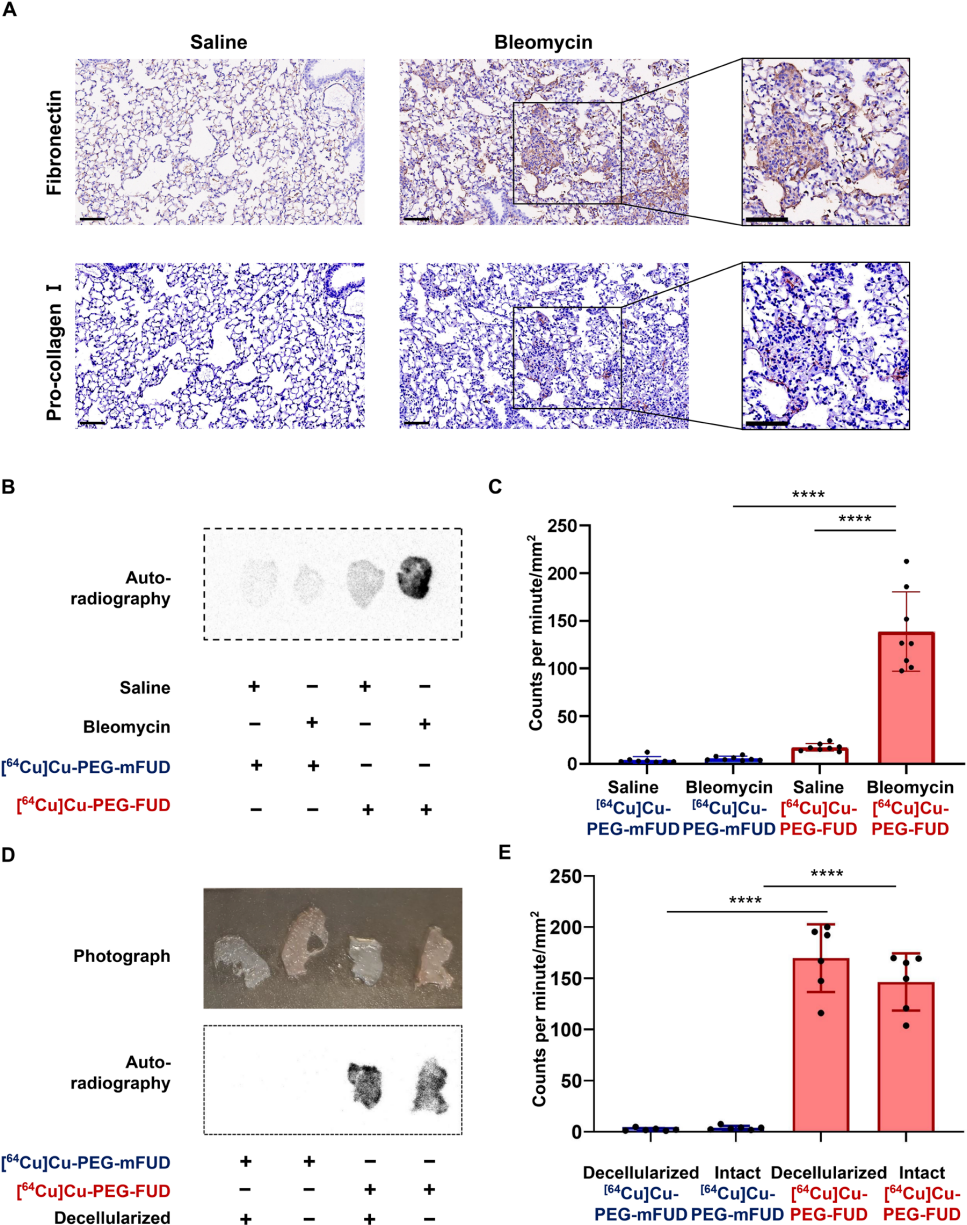
[^64^Cu]Cu-PEG-FUD targets the fibrotic mouse lung ex vivo. (**A**) Immunohistochemical staining against FN and procollagen in lungs collected 11 days posttreatment with saline or bleomycin. Scale bars, 100 μm. (**B**) Autoradiography of mouse PCLSs acquired from mouse lungs 11 days postsaline or bleomycin treatment and incubated with [^64^Cu]Cu-PEG-FUD or [^64^Cu]Cu-PEG-mFUD. (**C**) Normalized CPM by the area of tissues incubated with [^64^Cu]Cu-PEG-FUD or [^64^Cu]Cu-PEG-mFUD. One-way ANOVA with Šídák’s multiple comparisons test was used for statistical testing (****P < 0.0001). Data represent *n* = 4 mice per condition with duplicate sections per mouse and are depicted by scatterplot bar graphs with mean ± SD. (**D**) Photography and autoradiography of intact or decellularized PCLSs 11 days postbleomycin treatment and incubated with [^64^Cu]Cu-PEG-FUD [^64^Cu]Cu-PEG-mFUD. (**E**) Normalized CPM by the area of tissues incubated with [^64^Cu]Cu-PEG-FUD or [^64^Cu]Cu-PEG-mFUD. Each experiment was performed once in the laboratory with *N* ≥ 3 biological replicates for each experiment and *N* = 2 technical replicates within those in (B) to (E).

Before we proceeded with determining the capacity of [^64^Cu]Cu-PEG-FUD to bind murine fibrotic tissues, we confirmed the presence and localization of the fibrotic ECM. Immunohistochemical staining of the lung tissue revealed that FN (probe target) and procollagen I expression were increased 11 days after bleomycin treatment (Fig. 2A), during the profibrotic phase of the model, consistent with development of fibrosis and early collagen synthesis in the lungs (*23*, *48*). We observed that in saline-treated animals, alveolar architecture was normal, and there was scant staining of alveolar walls. In bleomycin-treated animals, high FN staining intensity was found in areas of thickened interstitium, consistent with areas of lung fibrosis. Procollagen staining was nearly absent in saline-treated control lungs, while staining was observed in areas of thickened interstitium of bleomycin-treated lungs, similar to the areas of FN stain.

Next, we set out to evaluate the specific binding of [^64^Cu]Cu-PEG-FUD to fibrotic lung tissues by performing ex vivo binding studies using living precision cut lung slices (PCLSs) obtained from mice 11 days posttreatment with bleomycin, when extensive profibrotic responses are developing (*46*). By using autoradiography and gamma counting with counts per minute (CPM) normalized to the area of the lung tissue, we found that [^64^Cu]Cu-PEG-FUD preferentially targets the fibrotic lung tissue in comparison to uninjured saline-treated controls and the [^64^Cu]Cu-PEG-mFUD control peptide (Fig. 2, B and C). The lack of binding of the [^64^Cu]Cu-PEG-mFUD control peptide indicated a negligible amount of nonspecific binding by the intact fibrotic tissue, supporting the specificity of PEG-FUD for FN-containing ECM. In addition, since PEG-FUD targets the N-terminal 70-kDa domain of FN, a key domain for cell-mediated FN matrix assembly, we also investigated whether [^64^Cu]Cu-PEG-FUD bound to cellular FN or decellularized FN matrix present in the lung tissues (*31*). Following decellularization, tissue color turned from pink to white (Fig. 2D, top), while the cell absence was also confirmed microscopically. After PCLS incubation with radiolabeled peptides, autoradiography and gamma counting demonstrated markedly higher radioactive signals in both intact and decellularized PCLSs incubated with [^64^Cu]Cu-PEG-FUD compared to those incubated with [^64^Cu]Cu-PEG-mFUD (Fig. 2, D and E), indicating that the specific binding sites for [^64^Cu]Cu-PEG-FUD remain despite loss of cellular content and further supporting the specificity of [^64^Cu]Cu-PEG-FUD targeting for FN-containing ECM.

### Biodistribution of [^64^Cu]Cu-PEG-FUD 3 days after bleomycin treatment

**Fig. 3.**
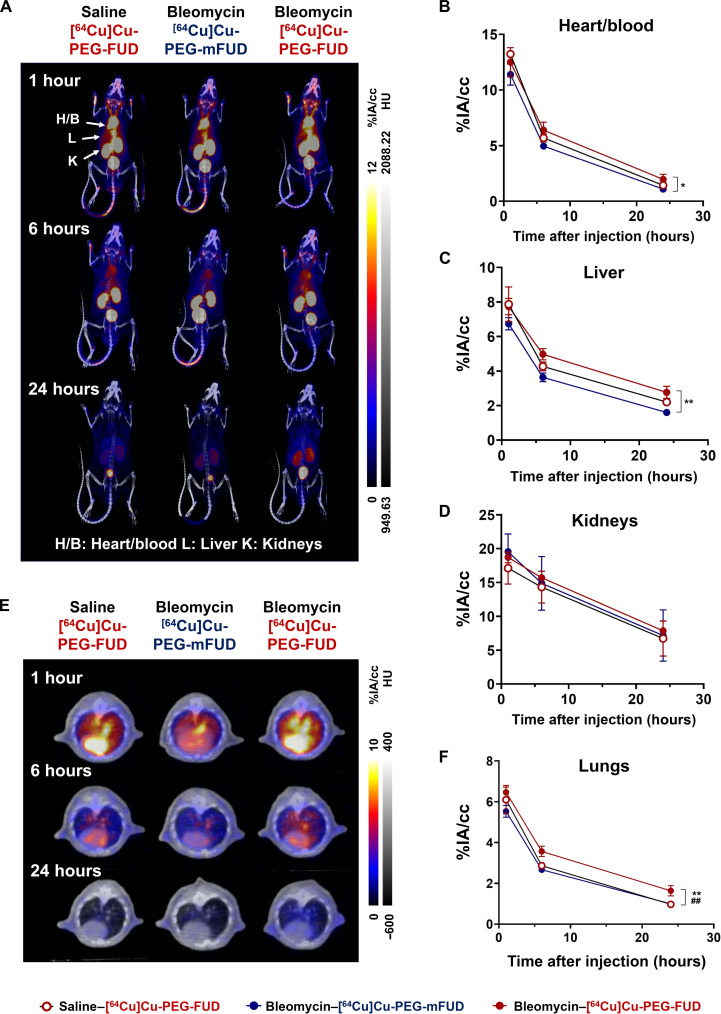
[^64^Cu]Cu-PEG-FUD targets mouse lungs 3 days postbleomycin injury. Mice were treated intratracheally with bleomycin (1 U/kg) or saline control 3 days before administration of low molar activity [^64^Cu]Cu-PEG-FUD or [^64^Cu]Cu-PEG-mFUD and subsequent sequential μPET/CT imaging. (**A**) Representative μPET/CT MIP and (**E**) axial images of saline- or bleomycin-treated mice acquired 1, 6, and 24 hours postinjection of [^64^Cu]Cu-PEG-FUD or [^64^Cu]Cu-PEG-mFUD. (**B**) Heart/blood, (**C**) liver, (**D**) kidneys, and (**F**) lung TAC of [^64^Cu]Cu-PEG-FUD or [^64^Cu]Cu-PEG-mFUD from longitudinal μPET/CT imaging. One-way ANOVA with Šídák’s multiple comparisons was used for statistical testing (*P < 0.05 between bleomycin–[^64^Cu]Cu-PEG-mFUD and bleomycin–[^64^Cu]Cu-PEG-FUD, **P < 0.01 between bleomycin–[^64^Cu]Cu-PEG-mFUD and bleomycin–[^64^Cu]Cu-PEG-FUD, and ##*P* < 0.01 between Normal Saline (NS)–[^64^Cu]Cu-PEG-FUD and bleomycin–[^64^Cu]Cu-PEG-FUD). Data represent *n* = 3 mice per condition within a single experiment and are depicted by decay curves representing mean ± SD.

To determine the spatiotemporal localization of PEG-FUD in vivo, we used the bleomycin-induced model of injury and fibrosis in mice combined with intravenous delivery of radiolabeled peptide and subsequent μPET/CT imaging. The bleomycin-induced pulmonary injury model is characterized by early inflammatory phase preceding the profibrotic phase (*46*, *49*) with FN deposition being apparent by day 3 postbleomycin treatment (fig. S3). In addition, since the FUD peptide binds to the N-terminal site of FN, a region that plays a critical role in initiating FN assembly, we sought to investigate the localization of [^64^Cu]Cu-PEG-FUD at this early time point following bleomycin injury. To confirm the nonspecific uptake of the probe due to inflammation and vascular leak at this stage, we used [^64^Cu]Cu-PEG-mFUD, a mutant control with greatly reduced affinity for FN (*37*, *43*). Given the physiological expression of FN in normal tissues, like the liver and lung and in circulation, FN is susceptible to antigenic sink effects that can detrimentally alter the biodistribution of [^64^Cu]Cu-PEG-FUD. A successful strategy to overcome the antigenic sink effects experienced by many targeted drugs such as monoclonal antibodies is to coadminister a “carrier” dose of the drug to saturate the binding sites in normal tissues and enhance its bioavailability and uptake in the target tissue (*50*–*52*). Our initial studies using noncarrier added (high molar activity) [^64^Cu]Cu-PEG-FUD demonstrated the FN sink effects, showing higher accumulation in the liver (fig. S4C), an organ synthesizing plasma FN (*53*). Furthermore, high molar activity of [^64^Cu]Cu-PEG-FUD exhibited lower imaging contrast in the lungs between the saline and bleomycin-treated groups at early imaging time points (fig. S4, E and F), plausibly attributed to the binding of the radiolabeled peptide to unsaturated plasma FN in both saline and bleomycin-treated groups. Thus, to reduce binding of [^64^Cu]Cu-PEG-FUD to plasma FN in the blood and nontargeting organs, a carrier dose of unlabeled PEG-FUD was coinjected with the radiolabeled (low molar activity) [^64^Cu]Cu-PEG-FUD (*25*, *54*) for our subsequent in vivo studies. When low molar activity of [^64^Cu]Cu-PEG-FUD was delivered 3 days postbleomycin instillation, maximum intensity projection (MIP) images and subsequent analysis of uptake in the heart/blood revealed its circulation in the blood up to 1-day postinjection and was higher than that of [^64^Cu]Cu-PEG-mFUD in bleomycin-treated mice (Fig. 3, A and B; *P* < 0.05). The average liver uptake in saline [2.2 ± 0.1 percent injected activity per cubic centimeter of tissue (%IA/cc)] or bleomycin-treated mice (2.8 ± 0.3%IA/cc) injected with [^64^Cu]Cu-PEG-FUD was significantly higher than that of [^64^Cu]Cu-PEG-mFUD at 24 hours (1.6 ± 0.1%IA/cc, *P* < 0.01, *n* = 3 per group; Fig. 3C), possibly in part due to the specific targeting of PEG-FUD to FN at the site of its production. In addition, we observed that both [^64^Cu]Cu-PEG-FUD and [^64^Cu]Cu-PEG-mFUD underwent renal elimination, which was confirmed by high accumulation of the radiotracers in the kidneys (Fig. 3D). By analyzing the axial images, we were able to visualize changes in [^64^Cu]Cu-PEG-FUD and [^64^Cu]Cu-PEG-mFUD heart and lung uptake 24 hours after the injection of radiotracers (Fig. 3E). The lung time-activity curve (TAC) in Fig. 3F confirmed higher average lung uptake of [^64^Cu]Cu-PEG-FUD in bleomycin-treated mice than all other control groups, including [^64^Cu]Cu-PEG-FUD in saline-treated mice or [^64^Cu]Cu-PEG-mFUD in bleomycin-treated mice as early as 6 hours (*P* < 0.01, *n* = 3 per group), suggesting its specific accumulation in the lung at day 3 posttreatment of bleomycin.

### Lung analysis of [^64^Cu]Cu-PEG-FUD 3 days after bleomycin treatment

**Fig. 4.**
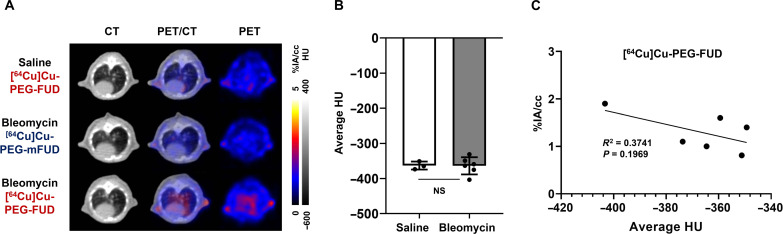
Early bleomycin-induced injury is detectable by [^64^Cu]Cu-PEG-FUD uptake via μPET imaging but does not result in alteration of μCT-detectable radiodensity. Mice were treated with a single intratracheal dose of bleomycin (1 U/kg) or normal saline. Three days later, mice were subjected to intravenous administration of low molar activity [^64^Cu]Cu-PEG-FUD or [^64^Cu]Cu-PEG-mFUD and μPET/CT imaging 24 hours postinjection. (**A**) Representative images of axial views are shown. (**B**) Average HU from μCT images of saline- or bleomycin-treated mouse lungs at 24 hours postinjection of radiotracers. Statistical analysis was performed using Student’s *t* test. NS, nonsignificant. Data represent *n* = 3 to 6 mice per condition within a single experiment and are depicted by scatterplot bar graphs with mean ± SD. (**C**) Correlation analysis using the average HU values from μCT imaging and %IA/cc values from μPET imaging identifying the level of [^64^Cu]Cu-PEG-FUD uptake in the lungs 24 hours postinjection. Linear regression was used for statistical analysis.

Following up on our initial analysis of the peptide biodistribution 3 days postbleomycin, we performed more detailed lung analysis using axial images acquired at 24 hours postinjection of radiotracers that showed an excellent imaging contrast after clearance of radiotracers from the heart (Fig. 4A). Given that HRCT is routinely used as one of the gold standard methods for assessing fibrotic burden and changes over time in the clinic and that μCT serves as a reliable means for assessment of lung fibrosis in the preclinical models of the disease (*55*–*57*), we chose to analyze μCT as a measure of overall fibrosis in our experiments. Three days after bleomycin treatment, there was no significant increase of radiodensity observed in the axial lung μCT images of the mice (*P* = 0.9755; Fig. 4B). Notably, while CT analysis did not reveal differences between saline- and bleomycin-treated mice 3 days postbleomycin, μPET images showed increased [^64^Cu]Cu-PEG-FUD uptake in the bleomycin-treated lung compared to saline-treated controls (Fig. 4A). In particular, μPET imaging analysis demonstrated significantly higher uptake (*P* < 0.01) of [^64^Cu]Cu-PEG-FUD in the bleomycin-treated lungs (1.63 ± 0.21%IA/cc) than in saline-treated lungs (0.97 ± 0.12%IA/cc). In addition, there was a significant difference between [^64^Cu]Cu-PEG-FUD uptake in bleomycin-treated lungs compared to that of the [^64^Cu]Cu-PEG-mFUD control peptide (0.99 ± 0.01%IA/cc) at 24 hours postinjection (*P* < 0.01, *n* = 3 per group; Figs. 3F and Fig. 4A). Given that [^64^Cu]Cu-PEG-mFUD has almost identical size and differs from [^64^Cu]Cu-PEG-FUD mainly by FN binding activity, the observed differential uptake of [^64^Cu]Cu-PEG-FUD compared to its control peptide was highly unlikely to be due to increased vascular permeability secondary to bleomycin lung injury or other mechanisms of nonspecific uptake. To investigate whether the lung accumulation of [^64^Cu]Cu-PEG-FUD correlated with the degree of lung tissue density at day 3 posttreatment with saline or bleomycin, we compared each animal’s average %IA/cc values from μPET imaging with the average Hounsfield unit (HU) values from μCT imaging obtained 24 hours after the injection of [^64^Cu]Cu-PEG-FUD. The linear regression analysis confirmed that there was no correlative relationship (*P* = 0.1969) observed between the μPET probe uptake (%IA/cc) and μCT radiodensity (HU) in saline- or bleomycin-treated mice administered [^64^Cu]Cu-PEG-FUD (Fig. 4C), suggesting that the specific accumulation of [^64^Cu]Cu-PEG-FUD in the lungs at this phase preceded the anatomical changes detectable by μCT.

### Biodistribution of [^64^Cu]Cu-PEG-FUD 11 days after bleomycin treatment

**Fig. 5.**
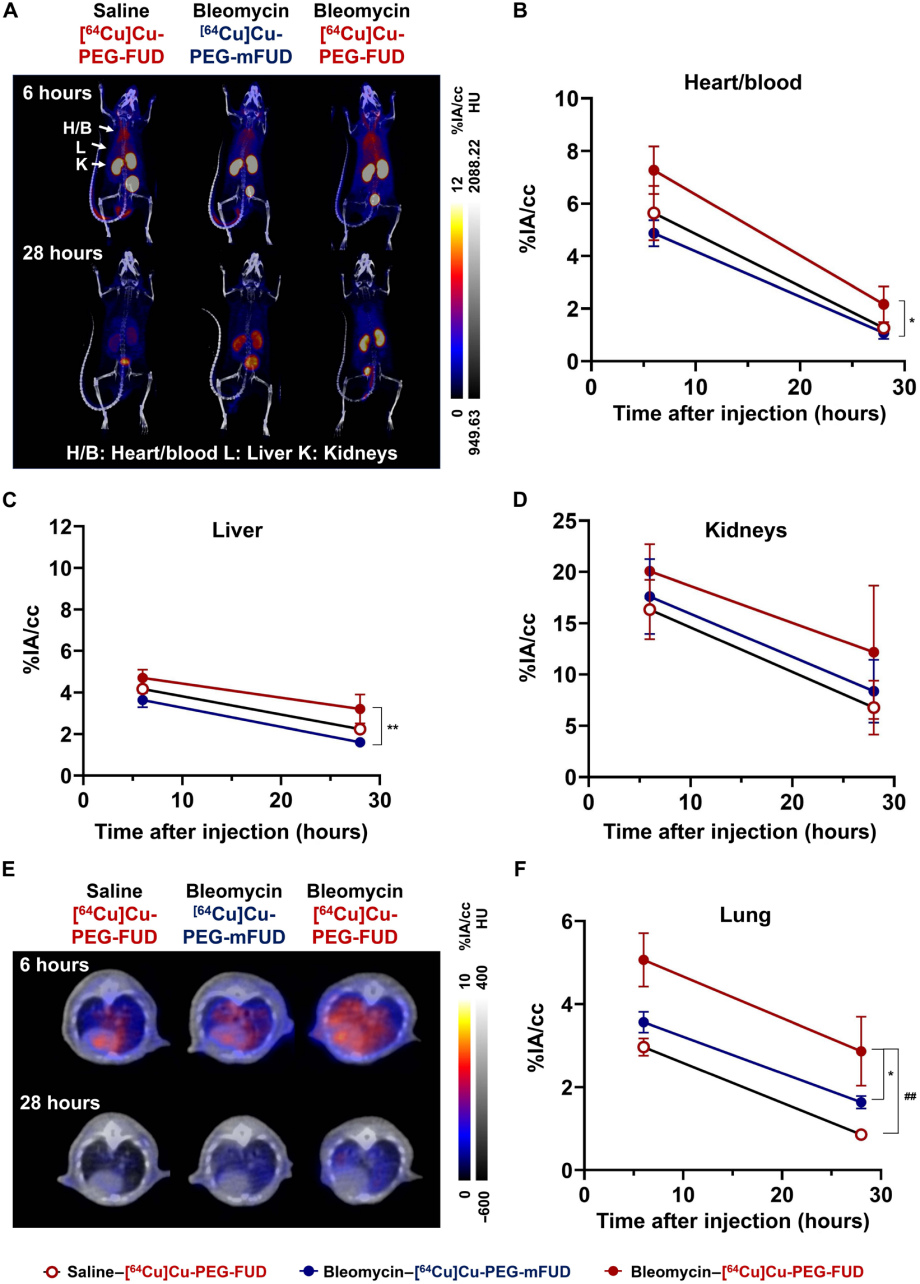
Detection of bleomycin-induced profibrotic phase via [^64^Cu]Cu-PEG-FUD μPET uptake. Mice were intratracheally treated with a single dose of bleomycin (1 U/kg) or saline control. Eleven days later, mice were administered a single low molar activity intravenous dose of [^64^Cu]Cu-PEG-FUD or [^64^Cu]Cu-PEG-mFUD control, followed by sequential μPET/CT imaging. (**A**) MIP and (**E**) axial images of μPET/CT imaging acquired 6 and 28 hours postinjection. TACs of (**B**) heart/blood, (**C**) liver, (**D**) kidneys, and (**F**) lung from μPET/CT imaging of [^64^Cu]Cu-PEG-FUD or [^64^Cu]Cu-PEG-mFUD injected into saline- or bleomycin-treated mice. One-way ANOVA with Šídák’s multiple comparisons was used for statistical testing (*P < 0.05 between bleomycin–[^64^Cu]Cu-PEG-mFUD and bleomycin–[^64^Cu]Cu-PEG-FUD, **P < 0.01 between bleomycin–[^64^Cu]Cu-PEG-mFUD and bleomycin–[^64^Cu]Cu-PEG-FUD, and ##*P* < 0.01 between NS–[^64^Cu]Cu-PEG-FUD and bleomycin–[^64^Cu]Cu-PEG-FUD). Data represent *n* = 3 mice per condition within a single experiment and are depicted by decay curves representing mean ± SD.

Given the encouraging findings 3 days postbleomycin injury, we next investigated the probe’s targeting to lung tissue during early profibrotic phase of the model. Thus, we delivered the radiolabeled probes 11 days postbleomycin treatment to the same mouse cohort previously imaged during the day 3 postbleomycin time point, followed by μPET/CT imaging. The MIP images from μPET/CT scans of mice after injection of [^64^Cu]Cu-PEG-FUD or [^64^Cu]Cu-PEG-mFUD are shown in Fig. 5A. The heart/blood accumulation of [^64^Cu]Cu-PEG-FUD was significantly higher than that of [^64^Cu]Cu-PEG-mFUD in bleomycin-treated mice (*P* < 0.05, *n* = 3 per group; Fig. 5B). Radiolabeled peptides in all groups exhibited a moderate level of liver uptake and a relatively high accumulation in the kidneys (Fig. 5, C and D), consistent with patterns shown in μPET/CT imaging performed 3 days postbleomycin. Furthermore, the liver uptake of [^64^Cu]Cu-PEG-FUD was significantly higher than that of [^64^Cu]Cu-PEG-mFUD (*P* < 0.01, *n* = 3 per group; Fig. 5C). The lung TAC analysis further confirmed that [^64^Cu]Cu-PEG-FUD peptide exhibited a significantly higher accumulation in bleomycin-treated lungs than in saline-treated lungs (*P* < 0.01, *n* = 3 per group), as well as in the lungs of mice treated with bleomycin and [^64^Cu]Cu-PEG-mFUD (*P* < 0.05, *n* = 3 per group) as early as 6 hours (Fig. 5F), confirming the specific targeting of [^64^Cu]Cu-PEG-FUD to bleomycin-treated lung tissues at day 11 posttreatment.

### Lung analysis of [^64^Cu]Cu-PEG-FUD 11 days after bleomycin treatment

**Fig. 6.**
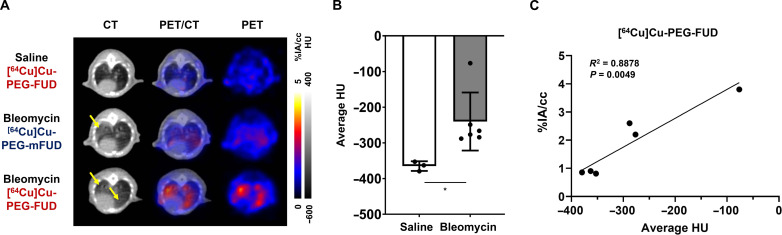
[^64^Cu]Cu-PEG-FUD PET lung uptake correlates with μCT-detected lung radiodensity in mice. Mice were treated with a single intratracheal dose of bleomycin (1 U/kg) or saline control, followed by intravenous administration of [^64^Cu]Cu-PEG-FUD or [^64^Cu]Cu-PEG-mFUD 11 days later and μPET/CT imaging 1 day thereafter. (**A**) Representative axial μCT, μPET/CT, and μPET images of saline- or bleomycin-treated mice at 28 hours postinjection of radiotracers. Yellow arrows indicate regions of increased radiodensity suggestive of fibrosis. (**B**) Average HU from μCT images of saline- or bleomycin-treated mice at 28 hours postinjection of radiotracers. Student’s *t* test was used for statistical testing (*P < 0.05). Data represent *n* = 3–6 mice per condition within a single experiment and are depicted by scatterplot bar graphs with mean ± SD. (**C**) Correlation analysis using the average HU values from μCT imaging and %IA/cc values from μPET imaging identifying the level of [^64^Cu]Cu-PEG-FUD uptake in the lungs 28 hours postinjection. Linear regression was used for statistical analysis.

We further analyzed the probe localization specific to the fibrotic lung during this early profibrotic phase of the model. Eleven days after treatment with bleomycin, the axial μPET/CT images from 28 hours postprobe administration imaging time were subsequently analyzed to determine the uptake of [^64^Cu]Cu-PEG-FUD and provide information about the development of fibrosis. Eleven days after the bleomycin treatment, during the high profibrotic response, we detected a significant increase (*P* < 0.05) in HU values of lung μCT images of mice, indicative of increased lung radiodensity due to the fibrotic responses (Fig. 6, A and B). PET analysis confirmed significantly higher lung uptake of [^64^Cu]Cu-PEG-FUD in bleomycin-treated mice than saline-treated controls (2.9 ± 0.7%IA/cc versus 0.9 ± 0.0%IA/cc, *P* < 0.01, *n* = 3 per group) at the 28-hour imaging time point (Figs. 5F and Fig. 6A). Moreover, we found an increased level of [^64^Cu]Cu-PEG-FUD uptake compared to [^64^Cu]Cu-PEG-mFUD (1.6 ± 0.1%IA/cc, *P* < 0.05) 28 hours after administration in bleomycin-treated mice, indicating that the increased signal from [^64^Cu]Cu-PEG-FUD at this time point was not due to nonspecific uptake but rather was related to the FN binding capacity of the probe. Moreover, these findings are consistent with results from ex vivo PCLS binding studies from day 11 after bleomycin lung injury (Figs. 2, B and C, 5F, and 6A). From the μPET/CT images, we observed that μPET signal from [^64^Cu]Cu-PEG-FUD in bleomycin-treated lungs was most notably increased in the animals with highest μCT-detected radiodensity (Fig. 6A). Thus, we assessed the relationship between the level of radiodensity in μCT imaging and probe uptake in μPET imaging in all mice treated with [^64^Cu]Cu-PEG-FUD. Correlation analysis confirmed that the average HU values and the average %IA/cc values obtained from [^64^Cu]Cu-PEG-FUD injected mice showed a strong positive correlation (*R*^2^ = 0.8878, *P* = 0.0049), suggesting that more [^64^Cu]Cu-PEG-FUD accumulated in the lungs with higher levels of μCT radiodensity (Fig. 6C).

### Ex vivo biodistribution of [^64^Cu]Cu-PEG-FUD 11 days after bleomycin treatment

**Fig. 7.**
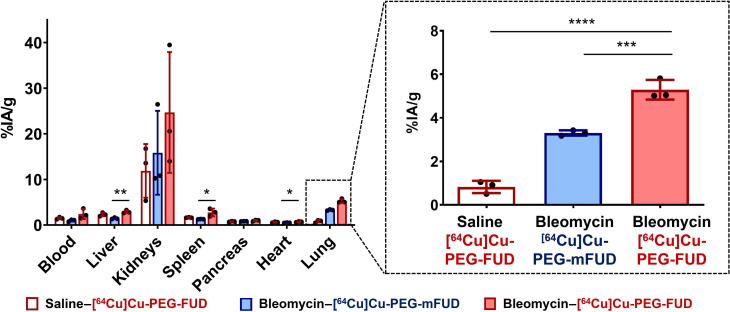
Ex vivo biodistribution of [^64^Cu]Cu-PEG-FUD corroborates the in vivo μPET-based detection of the radiolabeled probe. Eleven days after intratracheal treatment with bleomycin (1 U/kg) or saline control, mice were injected with [^64^Cu]Cu-PEG-FUD or [^64^Cu]Cu-PEG-mFUD, followed by ex vivo gamma counting of vital organs 1 day later. Dotted boxes show enlarged views of the average ex vivo lung uptake of each probe. One-way ANOVA with Šídák’s method for multiple comparisons was used for statistical testing (*P < 0.05, **P < 0.01, ***P < 0.001, and ****P < 0.0001). Data represent *n* = 3 mice per condition within a single experiment and are depicted by scatterplot bar graphs with mean ± SD.

Upon completion of day 11 imaging experiments, we measured radioactive signals in each organ via gamma counting to confirm biodistribution of radiolabeled peptides ex vivo. As shown in Fig. 7, the renal elimination was dominant for both [^64^Cu]Cu-PEG-FUD and [^64^Cu]Cu-PEG-mFUD, which is consistent with in vivo μPET/CT imaging results. In addition, the ex vivo lung uptake of [^64^Cu]Cu-PEG-FUD in bleomycin-treated lungs [5.3 ± 0.4 percent injected activity per gram of tissue (%IA/g)] was significantly higher than in saline-treated lungs (0.8 ± 0.2 %IA/g, *P* < 0.0001, *n* = 3 per group) or [^64^Cu]Cu-PEG-mFUD (3.3 ± 0.1 %IA/g, *P* < 0.001, *n* = 3 per group) in bleomycin-treated lungs, confirming the preferential and specific binding of [^64^Cu]Cu-PEG-FUD to bleomycin-treated lungs.

## DISCUSSION

There is an urgent clinical need for noninvasive real-time assessment of IPF disease activity with several targets being investigated for this purpose (*19*–*21*). FN plays a key role in the early development of pulmonary fibrosis as it is highly up-regulated in the fibroblastic foci and serves as a scaffold for deposition of other ECM proteins, including collagens, and correlates with IPF disease activity (*22*, *26*, *28*, *58*). Here, we used PEG-FUD peptide to target FN (*42*), revealing that it preferentially localizes to regions morphologically consistent with fibroblastic foci in the human IPF lung ex vivo and the early time points after bleomycin-induced lung injury in mice. These data suggest that PEG-FUD peptide has the potential to serve as noninvasive targeted PET probe for early detection of FN, which has been associated with disease activity.

IPF is characterized by spatial heterogeneity due to the simultaneous presence of nonfibrotic alveoli, fibroblastic foci, as well as mature (end-stage) collagen matrix in the same specimen (*22*). Fibroblastic foci represent the edge of progressive fibroproliferation with localization of pathogenic fibroblasts and myofibroblasts, differential up-regulation of FN, and collagen biosynthesis (*22*). Our ex vivo binding studies in human IPF lung samples demonstrate that Cy5-PEG-FUD in part colocalizes to structures resembling fibroblastic foci, providing a rationale to apply PEG-FUD peptide in detecting fibroblastic regions in lungs in vivo.

The present study used the single-dose bleomycin-induced mouse model of pulmonary fibrosis, a well-established model for preclinical studies of this disease (*47*, *59*), including for development of molecular imaging probes (*59*, *60*). This model is characterized by spatial heterogeneity in the bleomycin-treated mouse lung, providing an advantage due to its ability to mimic the spatial heterogeneity observed in human disease. During the profibrotic phase of the model (day 11 postbleomycin), radioactivity quantification in ex vivo living PCLS confirmed that [^64^Cu]Cu-PEG-FUD specifically binds to fibrotic lung tissue. The lack of binding activity of the [^64^Cu]Cu-PEG-mFUD control peptide further supports the specific binding of [^64^Cu]Cu-PEG-FUD to its targets in bleomycin-induced fibrotic lungs (*37*). The heterogeneous patterns of autoradiography of [^64^Cu]Cu-PEG-FUD in PCLSs imply that the binding of [^64^Cu]Cu-PEG-FUD may be relevant to the variegated expression of FN in fibrotic tissues that is specific to regions of nascent fibrogenesis (*47*, *61*). The binding of [^64^Cu]Cu-PEG-FUD was shown in both intact and decellularized tissues, suggesting that FN binding sites for [^64^Cu]Cu-PEG-FUD are available in fibrotic lung tissues regardless of cellular presence and activity. This result is also in line with our previous work showing that Cy5-PEG-FUD bound to both intact and decellularized matrix assembled by cancer-associated fibroblasts (*45*) and is consistent with the peptide’s binding specificity to deposited FN matrix.

Three days postbleomycin injury, [^64^Cu]Cu-PEG-FUD μPET imaging showed a significantly higher uptake in bleomycin-treated lungs than saline-treated controls, while μCT failed to distinguish between the normal and injured lungs. Early bleomycin injury results in vascular damage and increased capillary leakage that may permit enhanced permeability of molecular agents, including imaging probes. We used [^64^Cu]Cu-PEG-mFUD, a mutant control with a reduced affinity for FN, to investigate the basal probe uptake from potential increase of vascular leak during the inflammatory phase (*37*, *43*). We observed that the [^64^Cu]Cu-PEG-FUD uptake was significantly higher than the [^64^Cu]Cu-PEG-mFUD uptake, suggesting that [^64^Cu]Cu-PEG-FUD uptake at this time point is likely due to specific targeting to FN rather than nonspecific accumulation in the injured lungs. Evidence of increased FN-targeting probe uptake is consistent with previous reports showing an increase in FN content 3 days after the bleomycin treatment in the preclinical model (*62*) and our supporting evidence of microscopic injury and FN deposition at the same time point (fig. S3). Although additional studies are needed to further elucidate the mechanisms of this early probe uptake, given the critical role of FN in the development and progression of clinical IPF, our results suggest that sensitive and specific detection of FN changes at early stages of disease progression may serve in concert with other methods to provide clinical assessment of the disease and inform clinicians about disease progression.

μPET/CT imaging on day 11 postbleomycin treatment further confirmed the marked increase of [^64^Cu]Cu-PEG-FUD uptake in the profibrotic lungs compared to normal controls in vivo. We found that average μPET signal correlates with the average μCT radiodensity obtained from each mouse administered the [^64^Cu]Cu-PEG-FUD probe during this profibrotic phase of the model. Considering the subject-to-subject variability in this murine model of pulmonary fibrosis, we suspect that the animals exhibiting high levels of radiodensity by μCT and [^64^Cu]Cu-PEG-FUD uptake in μPET imaging may be experiencing high levels of fibrotic induction, suggesting that [^64^Cu]Cu-PEG-FUD probe may be useful as a predictor of disease progression and the developing fibrotic burden.

We found that molar activity of [^64^Cu]Cu-PEG-FUD affected its biodistribution. Liver uptake was more pronounced when [^64^Cu]Cu-PEG-FUD was injected at a high molar activity than when the radiotracer was spiked with unlabeled PEG-FUD. We suspect that codelivery of unlabeled PEG-FUD with [^64^Cu]Cu-PEG-FUD blocked a substantial portion of the plasma FN synthesized by the liver hepatocytes, acting as an antigen sink (*53*). [^64^Cu]Cu-PEG-FUD with reduced molar activity resulting from coadministration with unlabeled PEG-FUD still detected injured and fibrotic lungs in a sensitive manner, suggesting that the unlabeled PEG-FUD used in this study did not completely saturate its target in the lungs. In addition, [^64^Cu]Cu-PEG-FUD diluted with an unlabeled dose exhibited a high level of imaging contrast between the healthy and fibrotic lungs, indicating that an attempt to block circulating FN could have led to a higher degree of μPET signal attenuation in saline-treated lungs than in bleomycin-treated lungs, which are expected to contain more cellular FN deposited onto tissues due to the profibrotic responses (*29*, *45*, *63*, *64*).

Our study builds upon our previous work demonstrating the capacity of PEG-FUD to preferentially target 4T1 murine breast cancer tissue in mice, likely due to its targeting to high levels of FN in tumor tissues (*45*, *65*). As such, [^64^Cu]Cu-PEG-FUD probe may be able to serve more broadly to detect nascent FN deposition in other diseases characterized by ECM deposition. As in the previous study, we used a mutated FUD (mFUD) peptide with mutations to seven key FUD amino acids that results in reduction of FN binding (*37*). We found that the lung uptake of our control peptide, [^64^Cu]Cu-PEG-mFUD, did not show notable changes following bleomycin treatment, which is likely due to its compromised binding to FN in the lungs. The low level of uptake of [^64^Cu]Cu-PEG-mFUD evident in our μPET data may be a result of nonspecific accumulation or low level of partial binding of the mutant control to FN (*45*). However, the fact that lung uptake of [^64^Cu]Cu-PEG-FUD is significantly higher than [^64^Cu]Cu-PEG-mFUD on both day 3 and day 11 postbleomycin treatments supports that [^64^Cu]Cu-PEG-FUD accumulation in the lung is mainly due to specific binding of the peptide to FN rather than nonspecific uptake by vascular leakage or immune responses.

As already mentioned, the bleomycin-induced model of murine pulmonary injury and fibrosis is one of the most commonly used and well-accepted models for preclinical investigations related to IPF therapeutic (*57*, *66*, *67*) and diagnostic development (*19*, *20*, *30*, *59*) but is not without challenges. While the bleomycin-induced mouse model is similar to IPF disease in its spatial heterogeneity, it is not characterized by the presence of fibroblastic foci. For the purpose of this study, given the critical role of FN glycoprotein in fibroblastic foci, this is an important limitation. Thus, the application of these findings may require future utilization of larger animal models that are more representative of human disease. In addition, further studies using more progressive models of pulmonary fibrosis are needed to ascertain the utility of this peptide as a probe for disease activity (*68*). Furthermore, although we have previously shown that a 7-day course of PEG-FUD treatment resulted in a decrease in leukocyte infiltration in the kidney and did not show any sign of toxicity or distress (*43*), further investigation on the toxicity and immunogenic properties of this peptide will be performed in future studies. Last, although ^64^Cu radiolabel is appropriate for early preclinical studies, such as the one we are presenting here, the probe radiolabel will require further optimization to enable future clinical transability.

Now, IPF disease activity can only be assessed retrospectively via longitudinal changes in HRCT by detection radiographic opacities which are consistent with fibrotic changes and/or longitudinal changes in spirometric measurements such as forced vital capacity or diffusion capacity. Changes in these measures over 6- to 12-month time intervals correlate with disease progression. For this reason, PET-based biomarkers, such as this one, hold promise for improving clinical decision-making, by potentially working together with the existing methods to allow noninvasive, real-time assessment of disease activity for patients with IPF. This information would enable clinicians to expeditiously determine whether disease is “active,” which may guide the decision to initiate treatment and/or to determining the efficacy of clinically used or even investigational therapeutics for IPF. To this effect, future studies with [^64^Cu]Cu-PEG-FUD can be expanded to the detection of fibrotic areas in the lungs in more advanced stages of fibrosis and the capacity of [^64^Cu]Cu-PEG-FUD to predict future fibrotic burden or monitor the treatment responses after antifibrotic therapy. In addition, further optimization of molar activity or imaging time points may help the development of radiolabeled PEG-FUD as a PET probe for diagnosis of pulmonary fibrosis in a clinical setting.

## MATERIALS AND METHODS

### Study design

Our studies were designed to test the hypothesis that the FN-targeting peptide, PEG-FUD, can serve as an imaging probe for detection of pulmonary fibrosis. Therefore, we first performed observational studies to determine the binding potential and localization of fluorescently labeled PEG-FUD (Cy5-PEG-FUD) to IPF human tissue ex vivo, followed by assessment of radiation emitted by radio-labeled PEG-FUD ([^64^Cu]Cu-PEG-FUD) after binding to normal and fibrotic mouse lung sections. Next, we prospectively designed an in vivo study aimed at quantifying the uptake of [^64^Cu]Cu-PEG-FUD by PET imaging during the early phases of bleomycin-induced pulmonary inflammation and fibrosis in mice. Our sample size power calculations were based on previous determination of ECM content in response to bleomycin (*69*). Assuming a correlation of PET signal to ECM content, to obtain an 80% power to detect a twofold increase in signal at a *P* < 0.05, we found a requirement of *N* ≥ 3 mice per group. This study involved intravenous delivery of [^64^Cu]Cu-PEG-FUD to the same mouse cohort 3 and 11 days postbleomycin delivery, followed by incremental in vivo μPET/CT imaging up to 1 day postinjection and ex vivo gamma counting of vital organs. Mice were randomly selected for group placement. Immunohistochemical and image analysis was performed or confirmed by investigators blinded to the treatment groups. The amount of peptide conjugates was presented as peptide-equivalent.

### Immunohistochemistry

Human IPF tissue samples were obtained from thoracic surgical resection specimens from patients undergoing surgical biopsy or lung explant tissue from patients undergoing lung transplantation. Tissues were obtained by the University of Wisconsin-Madison Carbone Cancer Center Translational Science BioCore (Institutional Review Board approval no. 2011-0840). Histologic assessment was performed to verify the presence of the UIP architectural pattern for fibrotic tissue. Deidentified tissue samples only revealing diagnosis were then released to the research team under a research protocol (Institutional Review Board application no. 2011-0521) that was deemed nonhuman subjects research by the local Institutional Review Board and thus did not require the research team to obtain informed consent. Tissue samples were embedded in Tissue-Tek optimal cutting temperature compound (Sakura Finetek, Torrance, CA) and frozen before being sectioned to 8-μm thickness. Sequential lung tissue sections were subsequently subjected to staining with fluorescently tagged (Cy5)-PEG-FUD, Cy5-PEG-mFUD, and Masson’s trichrome and immunostaining against FN [rabbit polyclonal antibody, previously described (*70*)]. Staining with Cy5-PEG-FUD and mutant control was performed on nonfixed thawed tissue slices by blocking in 4% bovine serum albumin (BSA) in phosphate-buffered saline (PBS) for 30 min at room temperature (RT). Only for blockade studies, tissues were incubated with 375-μg FUD peptide (75× excess). In all experiments, tissues (either straight from incubation with 4% BSA or after the FUD blockade step) were incubated with 5 μg (peptide-equivalent) of Cy5-PEG-FUD or Cy5-PEG-mFUD diluted in 1% BSA in PBS for 60 min at RT in the dark. Tissue sections were subsequently washed and fixed with 10% formalin (Formal Fixx, Thermo Fisher Scientific, Waltham, MA) before being stained with 4′,6-diamidino-2-phenylindole (DAPI; 0.1 μg/ml, Tocris Bioscience, Bristol, UK), and coverslips were mounted with Prolong Gold antifade mounting medium (Invitrogen, Thermo Fisher Scientific). Slides were imaged by Histowiz Inc. using PhenoImager HT (Akoya Biosciences, Marlborough, MA) or Leica Thunder Imaging System (Leica Biosystems, Wetzlar, Germany) to visualize DAPI and Cy5, and snapshots or full tissue images were acquired in Phenochart (Akoya Biosciences) or Adobe Photoshop (San Jose, CA), respectively. Multiple images (*N* = 14) from a section corresponding to either nascent (*N* = 5) or mature fibrosis (*N* = 9) were isolated in the Masson’s trichrome stains of IPF tissue by an observer with expertise in IPF who was blinded to the Cy5-PEG-FUD and FN signal localization. The classification of fibrotic regions was further confirmed by an IPF clinician. In these stains, mature fibrosis was detected by heavy blue staining (outlined in white in fig. S1B), while structures consistent with fibroblastic focus morphology (representative of nascent fibrosis) were detected by their characteristic appearance of spindle-shaped cell aggregates found at the air-tissue interfaces (outlined in yellow in fig. S1B). For subsequent analysis (fig. S1, E and F), the regions of nascent and mature fibrosis identified in Masson’s trichrome stains were located and quantified in FN and peptide stains from consecutive IPF lung tissue sections (fig. S1, A to C). One representative image of nascent fibrosis is shown in Fig. 1 (A to D).

ImageJ was used to directly quantify fluorescence from Cy5-PEG-FUD staining, while the immunohistochemistry Toolbox was used to isolate the brown FN stain, followed by image inversion and measurement of integrated density (*49*, *57*, *71*). Masson’s trichrome and FN chromogenic stains were imaged using the Aperio Digital Pathology Slide Scanner System, and snapshots were acquired in Aperio ImageScope (Leica Biosystems).

Immunohistochemical staining of saline- or bleomycin-treated lungs was performed as reported previously with slight modifications (*57*). Briefly, mouse lungs were inflated and fixed with 10% formalin (Fisherbrand, Pittsburg, PA) and embedded in paraffin for sectioning. Serial lung sections were stained against FN [1:10,000; rabbit polyclonal antibody; previously described (*70*)] or procollagen I (1:100; rat monoclonal antibody; Abcam, ab64409, Cambridge, UK). The secondary antibody corresponding immunoglobulin G isotype was used for negative control staining. Stained slides were scanned via the Aperio Digital Pathology Slide Scanner System up to ×40 magnification and processed using Aperio ImageScope (Leica Biosystems, Wetzlar, Germany). To quantify the FN immunostaining, an average of ~77 ± 19 snapshots were taken of the lung parenchyma per mouse in *N* = 4 to 7 mice per condition. Magnification of ×20 and outlining of lung parenchymal regions were used to exclude blood vessels and airways so that FN expression could be quantified only in the parenchymal region of the lung. ImageJ immunohistochemistry Toolbox was used to isolate the brown stain, followed by inverting images and measuring of integrated density (*49*, *57*, *71*).

### [^64^Cu]Cu-PEG-FUD preparation

PEG-FUD, PEG-mFUD, and their 1,4,7-triazacyclononane-1,4,7-triacetic acid (NOTA) conjugates (hereafter called NOTA-PEG-FUD and NOTA-PEG-mFUD, respectively) were prepared as reported previously (*42*, *45*). Briefly, FUD and mFUD peptides were recombinantly overexpressed using pET-ELMER constructs (*32*, *37*) and N-terminally PEGylated via reductive amination chemistry. The peptides at a concentration of 2 mg/ml in PBS were mixed with *p*-SCN-Bn-NOTA (Macrocyclic) with a 1:1 molar ratio in NaHCO_3_ (pH 8.6) for 2 hours at RT. The mixture was purified using the PD-10 column (Cytiva). The radionuclide ^64^Cu was produced by the Cyclotron group at University of Wisconsin-Madison. For ^64^Cu labeling, NOTA-PEG-FUD and NOTA-PEG-mFUD were diluted in 300 μl of 0.1 M sodium acetate buffer (pH 5.5) and mixed with ^64^Cu at 5 nmol/mCi ratio (high molar activity: 5 to 10 GBq/μmol) for 1 hour at 37°C. Unlabeled ^64^Cu was removed via PD-10 column. The radiolabeling yield of ^64^Cu-labeled PEG-FUD ([^64^Cu]Cu -PEG-FUD) and PEG-mFUD ([^64^Cu]Cu -PEG-mFUD) was measured by TLC using 50 mM EDTA as a mobile phase and quantified to be >90%. Low molar activity (0.3 to 0.6 GBq/μmol) [^64^Cu]Cu -PEG-FUD was prepared by adding a carrier dose of unlabeled PEG-FUD to the radiolabeled peptide.

### Bleomycin-induced murine pulmonary injury and fibrosis

All animal experiments were approved by the University of Wisconsin-Madison’s Institutional Animal Care and Use Committee (protocol numbers M005823 and M005532) and done according to Animal Research: Reporting of In Vivo Experiments (ARRIVE) guidelines. Male and female mice (13 ± 3 weeks of age) were derived from an in-house colony on C57Bl/6J background or purchased from Jackson laboratories. Mice were anesthetized by intraperitoneal administration of ketamine (100 mg/kg; Zoetis Inc., Parsippany-Troy Hills, NJ) and xylazine (15 mg/kg; Akorn Pharmaceuticals, Lake Forest, IL) before a single intratracheal administration of bleomycin (1 U/kg; Teva Pharmaceutical Industries, Israel) dissolved in 50 μl of 0.9% NaCl irrigation (saline; Baxter, Madison, WI) or saline alone for non-injured controls. Bleomycin- and saline-treated animals were kept in separate cages throughout the course of the experiment.

### Ex vivo [^64^Cu]Cu-PEG-FUD binding studies

PCLSs collected from mice with experimental lung fibrosis (or normal saline-treated controls) were used to confirm the lung tissue binding of [^64^Cu]Cu-PEG-FUD and[^64^Cu]Cu-PEG-mFUD ex vivo. Eleven days after the saline or bleomycin treatment, mouse lungs (*n* ≥ 3) were inflated with a 1:1 mixture of 2% low-melt agarose (IBI Scientific, Dubuque, IA), and culture medium was composed of phenol red free Dulbecco’s modified Eagle’s medium + l-glutamine (Corning Inc.), streptomycin (100 μg/ml), amphotericin B (250 ng/ml), penicillin [100 U/ml; Penicillin-Streptomycin-Amphotericin (PSA), Corning Inc.], ciprofloxacin (10 μg/ml; Corning Inc.), and 2 mM l-glutamine (Corning Inc.) supplemented with 10% fetal bovine serum (HyClone, Buckinghamshire, UK). Following inflation, mouse lungs were immediately submerged in ice-cold culture medium and remained on ice until being sectioned using a vibratome (Leica VT1200S) to 200-μm thickness at 0.36 mm/s speed and 1.10-mm amplitude. PCLSs were subsequently incubated in culture media at 37°C for at least 30 min before all sectioning was complete and subsequent treatment could proceed. From each mouse, groups of consecutive PCLSs of similar sizes were subjected to different treatments, including, in some cases, half being decellularized while the remaining (intact) slices were kept in culture media. Tissue decellularization was performed on the basis of a previously described protocol with all steps being done at 37°C while rocking, unless noted otherwise (*72*). PCLSs were incubated in warm decellularization solution (8 mM CHAPS, 1 M NaCl, and 25 mM EDTA in PBS) for 30 min and subsequently washed three times in Dulbecco's Phosphate Buffered Saline (D-PBS) (Corning Inc.). Next, the sections were incubated in benzonase buffer [50 mM tris-HCl, BSA (0.1 mg/ml), and 1 mM MgCl_2_ in PBS] for 15 min while rocking, followed by incubation in benzonase solution [benzonase (50 U/ml) in benzonase buffer] for 1 hour. The sections were subsequently rinsed twice in rinsing solution [penicillin (1200 U/ml), streptomycin (1200 μg/ml), and amphotericin B (500 ng/ml) in PBS] for 30 to 60 min and then moved to culture media for 45 min.

The PCLSs (intact or decellularized) were placed in 12-well plates and incubated with 5 μCi (~30 nM) of either [^64^Cu]Cu -PEG-FUD or [^64^Cu]Cu -PEG-mFUD in 1 ml of culture media for 2 hours at 37°C. After incubation of radiolabeled peptides, the PCLSs were washed in PBS four times to remove unbound radiolabeled peptides. The radioactivity remaining in each tissue slice was measured by the gamma counter (PerkinElmer), and CPM were normalized by the area of each PCLS estimated using ImageJ software (*71*). To visualize the tissue binding of radiolabeled peptides to PCLSs, consecutive tissues receiving different treatments were mounted onto individual glass slides, cover-slipped, and subjected to autoradiography per the manufacturer’s protocol (PerkinElmer).

### μPET/CT imaging and biodistribution studies

μPET/CT imaging of [^64^Cu]Cu-PEG-FUD or [^64^Cu]Cu-PEG-mFUD was performed in the Small Animal Imaging and Radiotherapy Facility using the Inveon small-animal μPET/CT scanner (Siemens Medical Solutions, USA). The following parameters were used for CT scans: 80 kVp, 1000 μA, and 250 ms exposure time. Three or 11 days after the saline or bleomycin treatment, 5 to 10 MBq of low molar activity [^64^Cu]Cu-PEG-FUD or [^64^Cu]Cu-PEG-mFUD was injected intravenously into each mouse. High molar activity radiotracers were used to evaluate the effects of molar activity in saline- or bleomycin-treated mice (*n* = 3 to 5 per group). Static longitudinal μPET/CT images were acquired 1 hour, 4 to 6 hours, and up to 1 day (26 to 28 hours) postinjection of radiolabeled peptides. After in vivo imaging was completed, mice were euthanized, and their major organs were collected for ex vivo biodistribution studies using the gamma counter (PerkinElmer). Biodistribution data were analyzed in %IA/g.

### μPET/CT image analysis

The μPET/CT imaging analysis was performed using the vendor software, Inveon Research Workspace. Three-dimensional regions-of-interest (ROI) were selected for each organ, and the average μPET signal was presented as %IA/cc. For lung analysis, μPET/CT images were coregistered, and the lung ROIs were generated by an automatic thresholding tool in combination with manual drawing for the adjustment of fibrotic regions with high tissue density. The average HU and %IA/cc values obtained from each mouse were used for μPET-μCT signal correlation analysis. ROIs of all other organs (heart, liver, and kidneys) were analyzed using a manual drawing tool.

### Statistical analysis

Data were presented as mean ± SD. For comparison between two groups, two-tailed Student’s *t* test was used. One-way analysis of variance (ANOVA) analysis followed by Šídák’s multiple comparisons test was performed using GraphPad Prism 9.3 (Prism) for comparison between multiple groups. For μPET-μCT correlation analysis, linear regression method was used to fit the data. *P* values less than 0.05 were considered as statistically significantly different.
