# Supplementary material for: [64Cu]Cu-PEG-FUD peptide for noninvasive and sensitive detection of murine pulmonary fibrosis
Source: Sci Adv. 2024 Apr 10;10(15):eadj1444. doi: 10.1126/sciadv.adj1444 (PMC11006221; doi:10.1126/sciadv.adj1444)
Supplement: Supplementary file 1 — Figs. S1 to S4 [file sciadv.adj1444_sm.pdf]

Science Advances
AAAS

Supplementary Materials for

[^{64}Cu]Cu-PEG-FUD peptide for noninvasive and sensitive detection of murine pulmonary fibrosis

Hye Jin Lee *et al.*

Corresponding author: Ksenija Bernau, kbernau@medicine.wisc.edu

Sci. Adv. **10**, eadj1444 (2024)
DOI: 10.1126/sciadv.adj1444

This PDF file includes:

Figs. S1 to S4

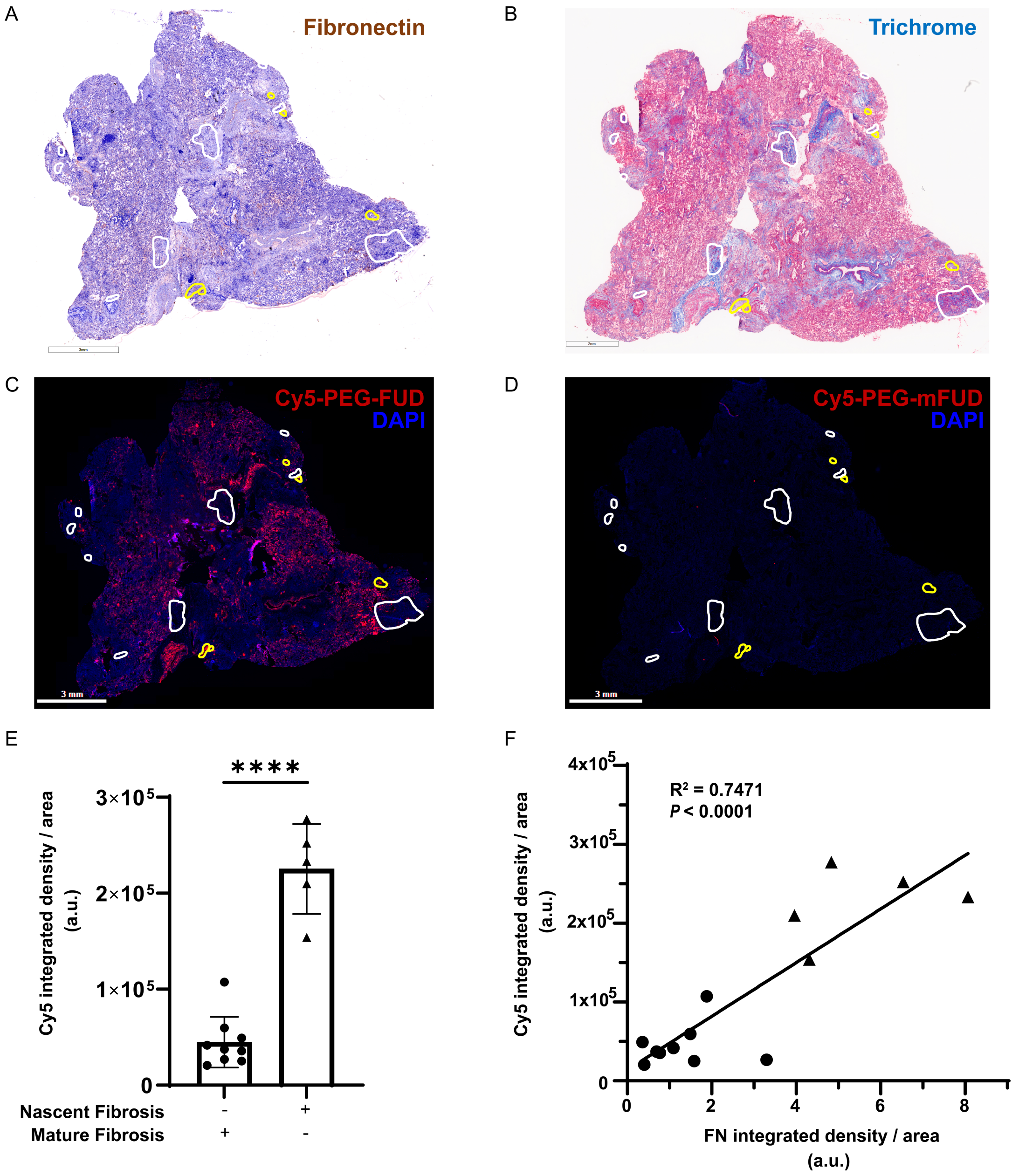

Fig. S1. **PEG-FUD targets fibronectin in regions of nascent fibrosis in IPF tissue *ex vivo*. (A)** Human IPF tissue was stained against fibronectin (FN) and **(B)** with Masson's trichrome stain. Tissue slices were incubated with **(C)** Cy5-labeled PEG-FUD (Cy5-PEG-FUD) or **(D)** mutant control (Cy5-PEG-mFUD), and DAPI as a nuclear stain. Scale bar = 3mm **(A, C, D)** and 2 mm **(B)**. Multiple regions depicting mature (white) and nascent fibrosis (yellow) were manually outlined in images from trichrome-stained sections **(B)**. The same areas were then located and outlined in images from Cy5-PEG-FUD stained-sections (**E, F**) or fibronectin IHC (**F**), followed by removal of nuclear (DAPI) signal and quantification of Cy5 integrated density or fibronectin (brown) integrated density (respectively). Circles = Mature Fibrosis, Triangles = Nascent Fibrosis. The experiment was performed once in the laboratory with N = 1 biological replicate. Data normalized to the area of each outline were analyzed using Student's *t*-test (****$p<0.0001$) or Pearson's correlation and were depicted by scatter plot or scatter plot bar graphs with mean ± SD.

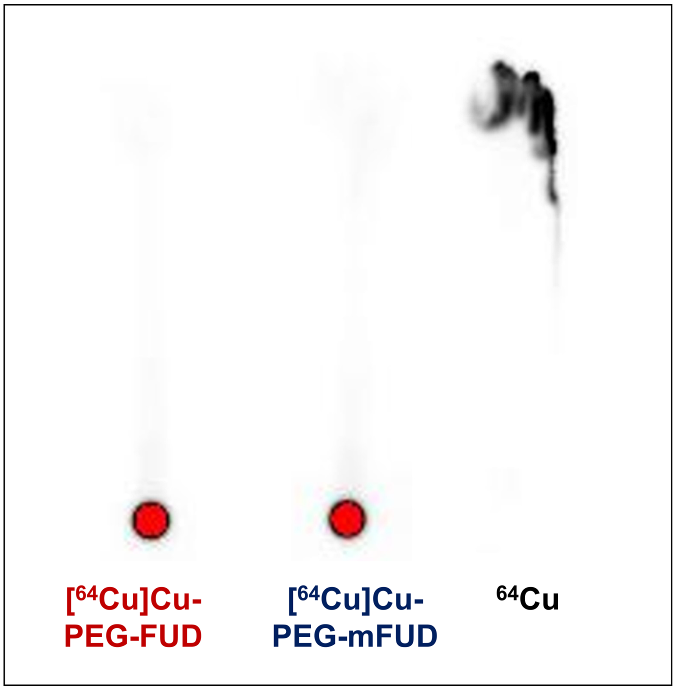

Fig. S2. Radio-thin layer chromatography (TLC) performed 60 min after radiolabeling of [^{64}Cu]Cu-PEG-FUD and [^{64}Cu]Cu-PEG-mFUD. Experiment was completed N ≥ 3 times.

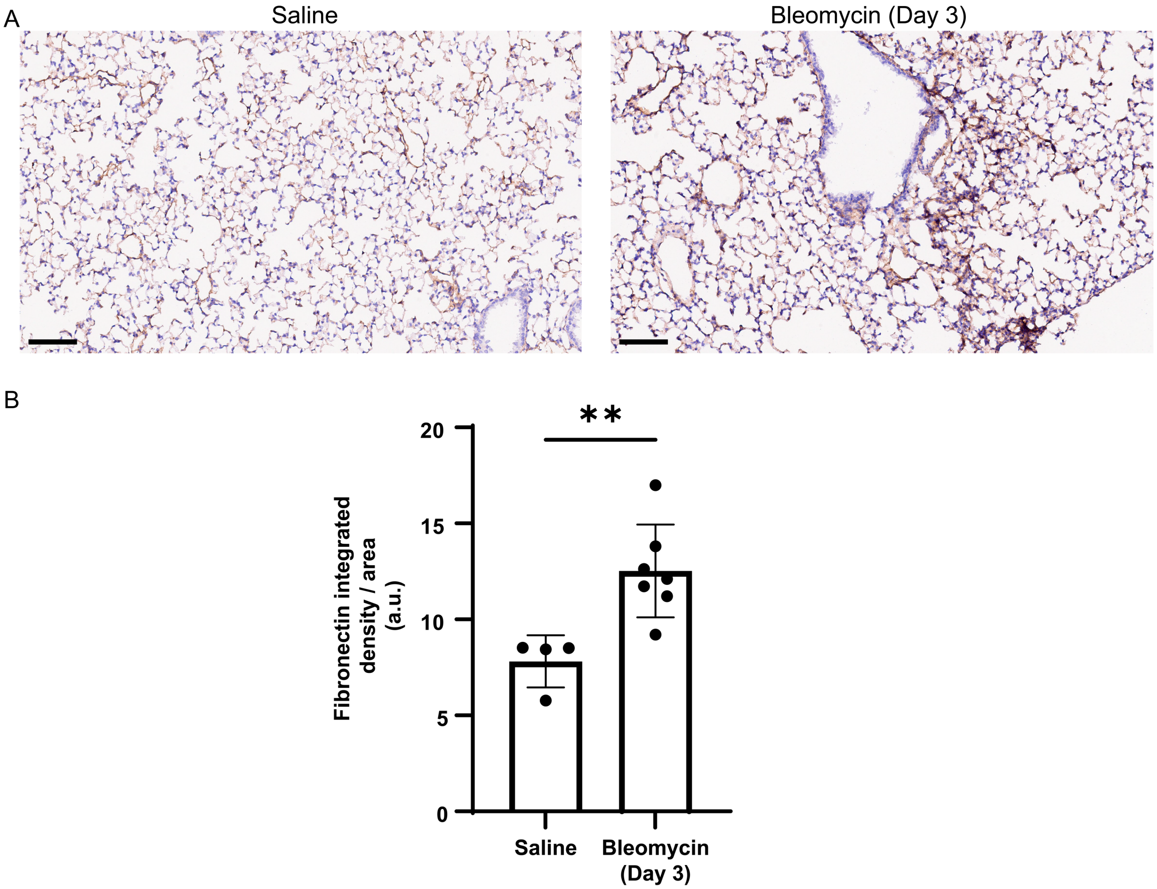

Fig. S3. **Immunohistochemistry confirms elevated fibronectin expression in bleomycin-treated lungs (A, right panel) than in saline-treated lungs (A, left panel) 3 days post-bleomycin treatment**. Scale bar = 100 µm. B. Fibronectin integrated density was quantified from IHC depicted in A. **: $P < 0.01$. The experiment was performed once in the laboratory with $N \geq 3$ biological replicates. Data are depicted by scatter plot bar graphs with mean ± SD.

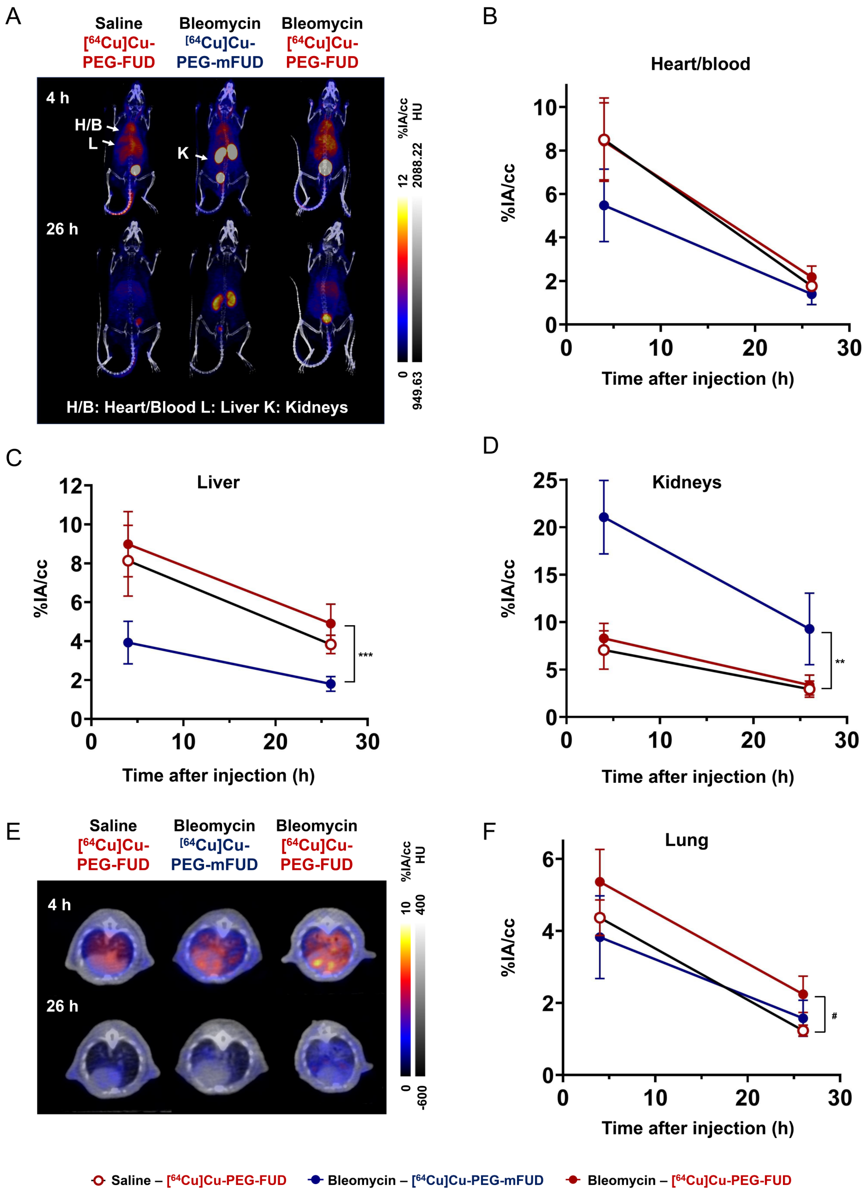

Fig. S4. **Biodistribution of [64Cu]Cu-PEG-FUD and [64Cu]Cu-PEG-mFUD injected without adding unlabeled doses**. Mice were intratracheally treated with bleomycin (1 U/kg) or saline control. Eleven days later, they were administered [64Cu]Cu-PEG-FUD or [64Cu]Cu-PEG-mFUD control without diluting with unlabeled dose, followed by sequential µPET/CT imaging. (A) Maximum Intensity projections (MIP) and (B) heart/blood, (C) liver, and (D) kidneys time-activity curves. (E) Representative axial images from µPET/CT imaging. (F) Lung time-activity curves obtained at 4 h and 26 h post-injection of radiotracers. %IA/cc: percent injected activity per cubic centimeter of tissue. One-way ANOVA with Šídák's multiple comparisons was used for statistical testing (#: $P < 0.05$ between NS–[64Cu]Cu-PEG-FUD and Bleomycin–[64Cu]Cu-PEG-FUD, **: $P < 0.01$ between Bleomycin–[64Cu]Cu-PEG-mFUD and Bleomycin–[64Cu]Cu-PEG-FUD, ***: $P < 0.001$ between Bleomycin–[64Cu]Cu-PEG-mFUD and Bleomycin–[64Cu]Cu-PEG-FUD. Data represent n = 3-5 mice/condition within a single experiment and are depicted by decay curves representing mean ± SD.
